# Improving biomedical named entity recognition with syntactic information

**DOI:** 10.1186/s12859-020-03834-6

**Published:** 2020-11-25

**Authors:** Yuanhe Tian, Wang Shen, Yan Song, Fei Xia, Min He, Kenli Li

**Affiliations:** 1 University of Washington, Seattle, USA; 2 Hunan University, Changsha, China; 3 The Chinese University of Hong Kong, Shenzhen, China; 4 Shenzhen Research Institute of Big Data, Shenzhen, China

**Keywords:** Named entity recognition, Text mining, Key-value memory networks, Syntactic information, Neural networks

## Abstract

**Background:**

Biomedical named entity recognition (BioNER) is an important task for understanding biomedical texts, which can be challenging due to the lack of large-scale labeled training data and domain knowledge. To address the challenge, in addition to using powerful encoders (e.g., biLSTM and BioBERT), one possible method is to leverage extra knowledge that is easy to obtain. Previous studies have shown that auto-processed syntactic information can be a useful resource to improve model performance, but their approaches are limited to directly concatenating the embeddings of syntactic information to the input word embeddings. Therefore, such syntactic information is leveraged in an inflexible way, where inaccurate one may hurt model performance.

**Results:**

In this paper, we propose BioKMNER, a BioNER model for biomedical texts with key-value memory networks (KVMN) to incorporate auto-processed syntactic information. We evaluate BioKMNER on six English biomedical datasets, where our method with KVMN outperforms the strong baseline method, namely, BioBERT, from the previous study on all datasets. Specifically, the F1 scores of our best performing model are 85.29% on BC2GM, 77.83% on JNLPBA, 94.22% on BC5CDR-chemical, 90.08% on NCBI-disease, 89.24% on LINNAEUS, and 76.33% on Species-800, where state-of-the-art performance is obtained on four of them (i.e., BC2GM, BC5CDR-chemical, NCBI-disease, and Species-800).

**Conclusion:**

The experimental results on six English benchmark datasets demonstrate that auto-processed syntactic information can be a useful resource for BioNER and our method with KVMN can appropriately leverage such information to improve model performance.

## Background

Biomedical named entity recognition (BioNER) is an important and challenging task for understanding biomedical texts. It aims to recognize named entities (NEs), such as diseases, gene, species, etc., in biomedical texts and plays an important role in many downstream natural language processing (NLP) tasks, such as drug-drug interaction task [21, 34] and knowledge base completion [38, 47]. Compared to named entity recognition in the general domain, BioNER is considered to be more difficult due to the lack of large-scale labeled training data and domain knowledge.

In the past decades, there have been many studies on BioNER, ranging from traditional feature based methods [4, 15–18, 20, 37] to recent deep learning based neural methods [5, 12, 19, 23, 32, 45]. Among the neural methods, the ones leveraging powerful encoders (e.g., biLSTM) achieve better results comparing with feature based methods because such encoders are good at modeling contextual information. More recently, pre-trained models such as ELMo [30] and BERT [6] achieved state-of-the-art performance on many NLP tasks in the general domain. Therefore, some studies [13, 19] applied them to BioNER yet found that these models cannot perform as well as in the general domain when there is no domain-specific information integrated. Therefore, Lee et al. [19] proposed a variant of BERT, namely, BioBERT, for the biomedical domain, which is pre-trained on large raw biomedical corpora and achieves state-of-the-art performance in BioNER.

**Fig. 1:**
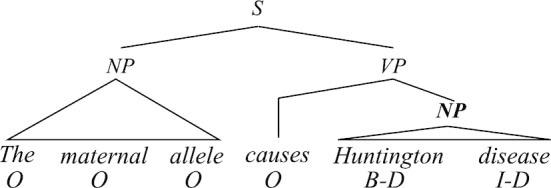
An example sentence. An example where the object noun phrase (“Huntington disease”) is a named entity. The labels under the words are BIO tags

In addition to the powerful encoders, syntactic information has also been playing an important role in many previous studies to help recognize biomedical named entities [4, 5, 20, 23, 37]. Intuitively, biomedical text often includes formal, well-structured, and long sentences, where syntactic information could be helpful because it can provide useful cues for recognizing NEs and thus help with the text understanding of NLP systems [36]. For example, Fig. 1 shows the parse tree of a sentence where the disease entity “Huntington disease” forms the object; thus, the boundary of a noun phrase can be a good cue for NER. Moreover, comparing with other types of extra resources, e.g., knowledge base [1, 24, 49], which are generally not publicly available or require human annotations, the syntactic information is easier to obtain through off-the-shelf NLP toolkits. Therefore, considering that the state-of-the-art BioBERT [19] does not leverage any syntactic information, we propose to improve BioBERT by incorporating the syntactic information of the input text, which is obtained from the parsing results of off-the-shelf toolkits.

To incorporate syntactic information into BioNER methods, previous studies have tried several ways. In the feature engineering methods, researchers use syntactic information to generate handcrafted features to help BioNER. For example, Song et al. [37] used part-of-speech (POS) and noun phrase tag features in a CRF-based BioNER system. In recent deep learning based methods, syntactic information is firstly represented by vectorized embeddings and then combined with word embedding through vector concatenation or element-wise summation, after which the resulting vector is fed into neural models to improve bioNER. For example, Luo et al. [23] used embedding vectors to represent syntactic information including POS and constituent labels, and concatenated those vectors with word embeddings. The combined embeddings were then sent into a biLSTM-CRF model with an attention mechanism to detect chemical NE. Dang et al. [5] proposed a model named D3NER, where the embeddings of various informative syntactic information are used to improve the results. Overall, previous approaches to leverage auto-processed syntactic information were limited to directly concatenating the embeddings of the syntactic information instances and the input words, without weighing the syntactic information instances in a specific context, where noisy information may hurt model performance. Therefore, we need to find a better method to leverage auto-processed syntactic information.

To weigh the syntactic information instances and leverage the important ones to improve BioNER methods, key-value memory networks (KVMN) [26] could be potentially useful, because it is demonstrated to be useful in leveraging extra information, e.g. knowledge base entities, to improve question answering tasks. In KVMN, the information is represented by key-value memory slots, where the keys are used to compute the weights for values by comparing these keys with the input, and the values are weighted summed according to the resulting weights and then used to make predictions. In addition, although the KVMN is originally proposed for question answering tasks, its variants also demonstrate impressive performance in many NLP tasks, such as Chinese word segmentation [40], semantic role labeling [11], and machine translation [27]. This motivates us to explore the possibility of using KVMN to leverage the syntactic information to improve BioNER.

Therefore, in this paper, we propose BioKMNER (KM stands for Key-value Memory network), which uses KVMN to incorporate syntactic information into the backbone sequence labeling tagger to improve BioNER. Specifically, we firstly use off-the-shelf toolkits to parse biomedical text sentences and extract three types of syntactic information: namely, POS labels, syntactic constituents, and dependency relations. Then, for each word in the input sentence, in the KVMN, we use the keys to represent the context features associated with the word and the values to denote the corresponding syntactic information instances. Therefore, context features (keys) are used to compute the weights by comparing them with the input word, and syntactic information instances (values) are weighed accordingly. Finally, the weighted summed values are concatenated with the output of the encoder, where the resulting vector is fed into the decoder for prediction. In this way, the method can incorporate the pair-wisely organized context features and syntactic information instances obtained from the toolkits simultaneously. Different from previous studies that directly use noisy syntactic information instances by embedding concatenation, our BioKMNER weighs them in KVMN and thus reduces the effect of error propagation caused by the noisy parsing results. We experiment BioKMNER on six English benchmark BioNER datasets covering four different entity types (i.e., chemical, disease, gene/protein, and species). The results demonstrate the effectiveness of our method for BioNER, where BioKMNER outperforms the BioBERT results reported by Lee et al. [19] on all datasets and achieves state-of-the-art results on four of them.

## Results

### Datasets

We evaluate our methods on six English benchmark datasets that are widely used in previous studies [10, 12, 16, 19, 48]. These datasets focus on four different biomedical entity types: BC2GM dataset [35] and JNLPBA dataset [14] for gene/protein NER, BC5CDR-chemical dataset [44] for chemical NER, NCBI-disease dataset [8] for disease NER, and LINNAEUS dataset [9] and Species-800 dataset [29] for species NER.

**Table 1:** The statistics of the four benchmark datasets

Datasets	Entity type		Token #	Sent. #	Entity #
BC2GM	Gene/protein	Train	355.4k	12.5k	15.1k
		Dev	71.0k	2.5k	3.0k
		Test	143.4k	5.0k	6.3k
JNLPBA		Train	443.6k	14.6k	32.1k
		Dev	117.2k	3.8k	8.5k
		Test	114.7k	3.8k	6.2k
BC5CDR-chemical	Chemical	Train	118.1K	4.5K	5.2K
		Dev	117.4K	4.5K	5.3K
		Test	124.7K	4.7K	5.3K
NCBI-disease	Disease	Train	135.7K	5.4K	5.1K
		Dev	23.9K	923	787
		Test	24.4K	940	960
LINNAEUS	Species	Train	281.2k	11.9k	2.1k
		Dev	93.8k	4.0k	711
		Test	165k	7.1k	1.4k
Species-800		Train	147.2K	5.7K	2.5K
		Dev	22.2K	830	384
		Test	42.2K	1.6K	767

“Token #”, “Sent. #” and “Entity #” represent the number of tokens, sentences, and entities

*BC2GM* BC2GM is a dataset used for the BioCreative II gene mention tagging task.^1^ It contains 20,000 sentences from abstracts of biomedical publications and is annotated with 24,583 mentions of the names of genes, proteins and related entities. It has become the major benchmark for the NER of gene/proteins entity type [10, 12, 19, 31, 43, 48].

*JNLPBA* JNPBA is the dataset for the Joint Workshop on NLP in Biomedicine and its Application Shared task.^2^ It was organized by the GENIA Project based on the annotations of the GENIA Term corpus and consists of 2404 publication abstracts. It is widely used for evaluating multi-class biomedical entity taggers.

*BC5CDR-chemical* BC5CDR is a dataset used for the BioCreative V Chemical Disease Relation (CDR) Task.^3^ It contains 1500 titles and abstracts from PubMed,^4^ where chemical and disease mentions are annotated by human annotators. Following previous studies [23], we only use the subset with chemical entities and denote it as BC5CDR-chemical.

*NCBI-disease* NCBI-disease contains 793 PubMed abstracts that are annotated with disease mentions and their corresponding concepts. There are 6,892 disease mentions from 790 unique disease concepts in this dataset and 91% of the mentions are mapped to a single disease concept. It has been a widely used research resource for the disease NER.

*LINNAEUS* The LINNAEUS dataset was created specifically for species named entity recognition and consists of 100 full-text documents. In the LINNAEUS dataset, all mentions of species terms were manually annotated and normalized to the NCBI taxonomy IDs of the intended species.

*Species-800* Species-800 is a novel benchmark corpus for species entities, which is based on manually annotated abstracts. It comprises 800 PubMed abstracts that contain identified organism mentions. Because the abstracts are select from journals on 8 different categories, the diversity of Species-800 is high and thus it is more challenging for NER systems.

We follow the study of Lee et al. [19] to pre-process all datasets. In details, BC2GM, BC5CDR-chemcial, LINNAUES, and NCBI-disease datasets are pre-processed based on the schema of Wang et al. [43]; JNPBA is pre-processed by CoNLL format;^5^ and Species-800 is pre-processed by Pyysalo.^6^ The statistics of all datasets in terms of the number of tokens, sentences, and entities are reported in Table 1.

### Implementation

In the experiments, we use off-the-shelf NLP toolkits to generate syntactic information, following the common practice in previous studies such as Mohit and Hwa [28], Tkachenko and Simanovsky [42], and Luo et al. [23]. In our study, we focus on three types of syntactic information: POS labels, syntactic constituents, and dependency relations. We use Stanford CoreNLP Toolkits (SCT)^7^ [25], which is a well-known NLP toolkit used in many studies [33, 39], to obtain the POS tagging, constituency, and dependency parsing results of a given input sentence.

For the encoder, considering that BERT [6] and its variants [2, 3, 7, 19] achieve state-of-the-art performance on many NLP tasks, we use the variant for the medical domain, i.e., BioBERT [19], in our method. Specifically, we use both the base and large version of BioBERT^8^ and follow the hyper-parameters used by Lee et al. [19] (i.e., for BioBERT-Base, there are 12 self-attention heads with 768-dimensional hidden vectors; for BioBERT-Large, the number of heads is 24 with 1024-dimensional hidden vectors). All parameters in the encoder are fine-tuned in training. For the KVMN module, the embeddings of all keys and values are randomly initialized, with their dimension matching the dimension of hidden vectors in the BioBERT encoder. Besides, we follow the setting of Lee et al. [19] to run the training process, where we do not use the “alternate” annotations for the BC2GM dataset. Moreover, for each method, we train five models with different random seeds to initialize the model parameters and use the average of their micro F1 scores for evaluation.^9^ In the experiments, we train each model for 150 epochs for the BC2GM dataset and for 60 epochs for all other datasets.^10^ In each run, we evaluate the model on the development set after each epoch to find its best performing result.

**Table 2:** Experimental results of models on six benchmark datasets

Methods	BC2GM		JNLPBA		BC5CDR-chemical		NCBI-disease		LINNAEUS		Species-800	
	F1	$\sigma$	F1	$\sigma$	F1	$\sigma$	F1	$\sigma$	F1	$\sigma$	F1	$\sigma$
Base	84.61	0.21	76.85	0.31	93.50	0.10	88.63	0.71	88.27	0.32	74.97	0.46
+ PL (DC)	84.47	0.15	*77*.*17*	0.45	93.66	0.15	89.09	0.55	88.36	0.16	75.04	0.46
+ PL (${\mathcal {M}}$)	$\mathit{84} .\mathit{74}$	0.10	77.06	0.05	$\mathit{93} .\mathit{73}$	0.19	$\mathit{89} .\mathit{47}$	0.56	$\mathit{88} .\mathit{44}$	0.30	$\mathit{75} .\mathit{45}$	0.41
+ SC (DC)	84.45	0.19	76.80	0.45	93.68	0.13	89.18	0.26	88.23	0.33	75.37	0.51
+ SC (${\mathcal {M}}$)	$\mathit{84} .\mathit{76}$	0.21	$\mathit{77} .\mathit{17}$	0.16	$\mathit{93} .\mathit{74}$	0.11	$\mathit{89} .\mathit{27}$	0.52	$\mathit{88} .\mathit{68}$	0.30	$\mathit{75} .\mathit{65}$	0.50
+ DR (DC)	84.33	0.30	77.01	0.28	93.66	0.15	89.05	0.23	88.43	0.19	75.12	0.52
+ DR (${\mathcal {M}}$)	$\mathit{84} .\mathit{65}$	0.27	$\mathit{77} .\mathit{32}$	0.35	$\mathit{93} .\mathit{78}$	0.18	$\mathit{89} .\mathit{24}$	0.60	$\mathit{88} .\mathit{57}$	0.15	$\mathit{75} .\mathit{81}$	0.71
Large	84.89	0.17	77.29	0.19	93.90	0.31	88.65	0.59	88.87	0.65	74.98	0.59
+ PL (DC)	85.06	0.08	$\mathit{77} .\mathit{56}$	0.18	93.90	0.16	88.74	0.26	88.65	0.39	74.92	0.86
+ PL (${\mathcal {M}}$)	$\mathit{85} .\mathit{07}$	0.12	77.50	0.19	$\mathit{94} .\mathit{05}$	0.23	$\mathit{88} .\mathit{86}$	0.29	$\mathit{89} .\mathit{01}$	0.31	$\mathit{75} .\mathit{34}$	0.95
+ SC (DC)	85.12	0.13	77.56	0.12	93.95	0.09	88.78	0.54	$\mathit{89} .\mathit{01}$	0.28	$\mathit{75} .\mathit{38}$	0.29
+ SC (${\mathcal {M}}$)	$\mathit{85} .\mathit{43}$	0.15	$\mathit{77} .\mathit{83}$	0.19	$\mathit{93} .\mathit{99}$	0.13	$\mathit{88} .\mathit{87}$	0.37	88.92	0.35	75.08	0.68
+ DR (DC)	85.01	0.12	77.58	0.10	93.97	0.17	$\mathit{89} .\mathit{37}$	0.30	88.99	0.22	75.01	0.83
+ DR (${\mathcal {M}}$)	$\mathit{85} .\mathit{17}$	0.10	$\mathit{77} .\mathit{73}$	0.11	$\mathit{94} .\mathit{05}$	0.10	88.81	0.51	$\mathit{89} .\mathit{04}$	0.27	$\mathit{75} .\mathit{17}$	0.91

The experimental results are reported in terms of average F1 scores (F1) and the standard deviation $\sigma$. The methods in the group “Base” and “Large” refer to baselines with BioBERT-Base and BioBERT-Large encoder and our methods with KVMN (${\mathcal {M}}$). “DC” refers to the baseline method using direct concatenation to incorporate syntactic information. “PL”, “SC”, and “DR” stand for POS labels, syntactic constituents, and dependency relations, respectively

### Overall results

We run the baseline methods without using syntactic information and our methods with KVMN (${\mathcal {M}}$) to incorporate three types of syntactic information obtained from SCT on six aforementioned datasets, where two different encoders, i.e., BioBERT-Base and BioBERT-Large, are used. For reference, we also run baseline methods that use direct concatenation (DC) to incorporate such syntactic information, where the embeddings of context features and syntactic information are directly concatenated with the output of the BioBERT encoder. We report the experimental results (the average F1 scores of the five runs for each method as well as the standard deviations $\sigma$) in Table 2. There are some observations.

First, comparing with the baseline methods without using any syntactic information, our method with KVMN can work well with both BioBERT-Base and BioBERT-Large encoder, where decent improvements over the baseline methods are observed among all datasets.

Second, compared with DC, our methods with KVMN to incorporate syntactic information achieve better results in most cases. For example, on the Species-800 dataset, our method (Base + DR (${\mathcal {M}}$)) obtains an average F1 score of $75.81\%$, while its corresponding DC-based method (Base + DR (DC)) obtains a lower average F1 score of $75.12\%$. Besides, in some cases where DC is applied, the syntactic information causes inferior results than baselines. For example, on the LINNAEUS dataset, the average F1 score of the DC-based method with the POS labels (Large + PL (DC)) is lower than the baseline (Large) results. One possible explanation could be: there are some noisy syntactic results extracted by off-the-shelf toolkits, which may influence the performance of the model and lead to worse results compared to the baselines only using BioBERT. Under this condition, methods with DC fails to distinguish the salient syntactic information that contributes more to the bioNER task in a specific context. On the contrary, KVMN can weigh such syntactic information according to the importance of the context features and thus, to some extent, avoid the errors caused by incorporating auto-processed syntactic information.

Third, in many cases, in methods with KVMN, the information of syntactic constituents (SC) and dependency relations (DR) offers higher improvement than POS labels (PL). For example, on the BC2GM dataset, our method with the BioBERT-Large encoder obtains the average F1 scores of $85.43\%$ and $85.17\%$ when it is enhanced by SC and DR, respectively, while its average F1 score is $85.07\%$ when PL is incorporated. One possible reason to explain the phenomenon could be: (1) the syntactic constituents can provide a cue of phrase functions and their boundaries (e.g., an NP treelet is not only a signal that can suggest there might be an NE inside, but also can give information about the possible starting and ending positions for that potential NE.); (2) the dependency relations link words in long-distance with their dependency relationships, which could be especially useful for biomedical texts that generally include long sentences and entities.

**Table 3:** Comparison with previous deep learning based methods

Methods	BC2GM	JNLPBA	BC5CDR-chemical	NCBI-disease	LINNAEUS	Species-800
biLSTM + pre-trained embeddings [12]	78.57	77.25	91.05	84.64	$\mathit{94} .\mathit{13}$	73.11
biLSTM + attentions [23]	–	–	92.57	–	–	–
biLSTM + multi-task learning [43]	80.74	73.52	-	86.14	–	–
biLSTM + pre-training [31]	81.69	75.03	–	87.34	–	–
biLSTM + transfer learning [10]	78.66	–	91.64	84.72	93.54	74.98
biLSTM + model ensemble [48]	79.73	$\mathit{78} .\mathit{58}$	93.31	86.36	–	–
SciBERT [3]	–	77.28	–	88.57	–	–
BERT [19]	81.79	74.94	91.16	85.63	87.60	71.63
BioBERT (Base) [19]	84.72	77.49	93.47	89.71	88.24	75.31
BioBERT (Large) [19]	85.01	–	–	88.79	–	–
BioBERT (Base) + DR (${\mathcal {M}}$)	84.92	77.72	94.00	$\mathit{90} .\mathit{08}$	88.79	76.21
BioBERT (Large) + DR (${\mathcal {M}}$)	$\mathit{85} .\mathit{29}$	77.83	$\mathit{94} .\mathit{22}$	89.63	89.24	$\mathit{76} .\mathit{33}$

The result (F1 scores) of our method on each dataset comes from the best performing model. The results for the base and large version of BioBERT [19] are from their paper and GitHub repository

We report the results of their version 1.1, which is identical to the BioBERT version used in our experiments

## Discussion

### Comparison with previous studies

We compare the results of our best performing model with previous studies on all aforementioned datasets. The results (F1 scores) are summarized in Table 3, where our method outperforms the previous study (i.e., Lee et al. [19]) using the base and large version of BioBERT on all datasets. Specifically, for the BioBERT-Base, the improvement of F1 scores on BC2GM, JNLPBA, BC5CDR-chemical, NCBI-disease, LINNAEUS, and Species-800 are $0.20\%$, $0.23\%$, $0.53\%$, $0.37\%$, $0.55\%$, and $0.90\%$ respectively; for the BioBERT-Large, the improvement on BC2GM and NCBI-disease are $0.28\%$ and $0.84\%$, respectively. These results demonstrate the effectiveness of our method to leverage auto-processed syntactic information in recognizing different types of named entities in the biomedical domain. In addition, our method achieves state-of-the-art performance on four datasets, i.e., BC2GM, BC5CDR-chemical, NCBI-disease, and Species-800. Compared with [48] and [12], we do not outperform their results on JNLPBA and LINNAEUS, because the gaps between their results and our baseline method, i.e., BioBERT from Lee et al. [19], are big on these datasets, which could be hard to compensate for by using syntactic information. Except for the two datasets, our method outperforms their methods on all other datasets.

### Effect of syntactic information ensemble

To explore the effect of using different types of syntactic information together, we conduct syntactic information ensemble experiments on the BC5CDR-chemical dataset. In the experiments, we test different combinations of different types of syntactic information, where two ensemble strategies are used. The first sums the weighted value embeddings of each type of syntactic information; and the second uses concatenation. The results of the average F1 scores of different settings are reported in Table 4, where the results form the baseline methods without using any syntactic information are also included for reference. We have several observations from it. First, overall, compared with the baseline methods, our methods achieve better results with both the base and large versions of the BioBERT encoder. This indicates that the combination of different types of syntactic information can help with the performance of the baseline method for BioNER. Second, the concatenation strategy performs better than the summing strategy in syntactic information fusion. One possible explanation could be: summing the embeddings of different types of syntactic information may lose some information while concatenating them can keep all information on all types of syntactic embedding.

**Table 4:** Results of the syntactic information ensemble on BC5CDR-chemical dataset

Ensemble strategies	Syntactic info.			BioBERT-Base		BioBERT-Large	
	PL	SC	DR	F1	$\sigma$	F1	$\sigma$
Baseline	$\times$	$\times$	$\times$	93.50	0.10	93.90	0.31
Sum	$\surd$	$\surd$	$\times$	93.66	0.17	94.20	0.15
	$\surd$	$\times$	$\surd$	93.76	0.16	94.10	0.15
	$\times$	$\surd$	$\surd$	93.81	0.15	94.12	0.14
	$\surd$	$\surd$	$\surd$	93.78	0.25	94.26	0.16
Concatenation	$\surd$	$\surd$	$\times$	93.75	0.23	94.25	0.12
	$\surd$	$\times$	$\surd$	93.80	0.26	94.22	0.16
	$\times$	$\surd$	$\surd$	93.83	0.20	94.31	0.08
	$\surd$	$\surd$	$\surd$	$\mathit{93} .\mathit{88}$	0.26	$\mathit{94} .\mathit{36}$	0.25

The three types of syntactic information used for the ensemble are POS labels (PL), syntactic constituents (SC), and dependency relations (DR). The results are reported in terms of the average F1 scores and the standard deviation ($\sigma$). Sum and concatenation are two ensemble strategies applied to our method

### Effect of different toolkits

To explore the effect of using syntactic information from different NLP toolkits, in addition to SCT, we try another toolkit, i.e., spaCy,^11^ to obtain the auto-processed syntactic information. In the experiments, we try two types of syntactic information, i.e., POS labels (PL) and dependency relations (DR), from the POS tagger and dependency parser of spaCy. We report the results (the average F1 scores and the standard deviation $\sigma$) of our methods with KVMN on the BC5CDR-chemical dataset in Table 5. For reference, the results of our method using SCT as well as the baseline results are also reported. From the results, we can find that, for both base and large BioBERT encoders, our method can leverage the syntactic information from different NLP toolkits and thus achieves better performance comparing with the baseline methods.

### Case study

**Fig. 2:**
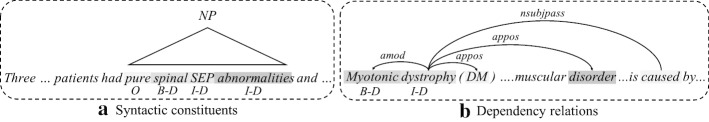
Case study. In the figure, **a**, **b** are two examples of syntactic information (i.e., syntactic constituents and dependency relations) and the context features for “SEP” and “dystrophy”, respectively. The weights for syntactic information learned from the memories are highlighted with the darker color referring to greater value

To better understand how our method improves BioNER, we conduct a case study where two example sentences are used. In Fig. 2a, b, we show two sentences and illustrate the way of syntactic constituents and dependency relations to improve bioNER, respectively. In both cases, for a specific word, we visualize the weights assigned to the corresponding syntactic information instances (values) on its associated contextual features (keys), where the deeper color refers to the higher weight.

**Table 5:** Results of using different NLP toolkits on the BC5CDR-chemical dataset

	BioBERT-base		BioBERT-large	
	F1	$\sigma$	F1	$\sigma$
Baseline	93.50	0.10	93.90	0.31
Stanford CoreNLP Toolkits				
PL (${\mathcal {M}}$)	93.73	0.19	94.05	0.23
DR (${\mathcal {M}}$)	$\mathit{93} .\mathit{78}$	0.18	94.05	0.10
spaCy				
PL (${\mathcal {M}}$)	93.69	0.12	$\mathit{94} .\mathit{06}$	0.10
DR (${\mathcal {M}}$)	93.71	0.12	93.97	0.13

The experimental results [the average F1 scores and the standard deviation ($\sigma$)] of our method with KVMN (${\mathcal {M}}$) using different NLP toolkits (i.e., Stanford CoreNLP Toolkits and spaCy) to obtain POS labels (PL) and dependency relations (DR). The results of baseline methods without using any syntactic information are also reported for reference

*Syntactic constituents* In the example sentence shown in Fig. 2a, the word we focus on is “SEP”. In this case, the constituent information firstly narrows the context features of “SEP” down to the words within the noun phrase “pure spinal SEP abnormalities”. Then, the KVMN module assigns the highest weight to “abnormalities” and its carrying syntactic information of “NP” among all other syntactic instances since they could be strong signals for disease names. Therefore, our method could assign the correct NE label to “SEP”. Likewise, the situation for “pure” is on the opposite and thus it receives the lowest weight among other words.

*Dependency relations* In addition, in Fig. 2b, we visualize the weights assigned to dependency relations for the word “dystrophy” in another example sentence. In this case, dependency information successfully finds the dependents, i.e., “Myotonic”, “DM”, and “disorder”, of “dystrophy”, which could suggest useful cues to predict the NE labels. Among those dependents, KVMN distinguishes that the dependent “discorder” with an “appos” dependency relation (appositional modifier) strongly suggests “dystrophy” is a disease entity. Therefore, KVMN assigns the highest weight to the dependency relation offered by “disorder”. Similarly, another modifier (i.e., “Myotonic”) of “dystrophy” is also distinguished and weighed by the KVMN, and the second-highest weight is assigned to it accordingly. It is worth noting that the dependency information that contributes most to recognizing “dystrophy” as a part of an NE comes from a word (“disorder”) in the long-distance; dependency information is able to capture that information and helps our method predict the NE tag for the word “dystrophy”.

## Conclusion

In this paper, we propose a method named BioKMNER with KVMN to enhance BioNER with auto-processed syntactic information (i.e., POS labels, syntactic constituents, and dependency relations) from off-the-shelf toolkits. In KVMN, context features and their corresponding syntactic information instances are mapped into keys and values, respectively. The values are weighed according to the comparison between the keys and the input words. Then the values are weighed summed and the resulting vector is fed back to the backbone tagging process to make predictions. In doing so, compared with previous studies that treat different syntactic information equally and leverage them by embedding concatenation, our method can discriminatively leverage the auto-processed syntactic information and avoid the error spread caused by the direct use of auto-processed syntactic results. The experimental results on six English benchmark datasets demonstrate that syntactic information can be a good resource to improve bioNER and our method with KVMN can appropriately leverage such information. In addition, our method outperforms the strong baseline method from the previous study using BioBERT [19] on all datasets and achieves state-of-the-art results on BC2GM, BC5CDR-chemical, NCBI-disease, and Species-800 datasets.

## Methods

**Fig. 3:**
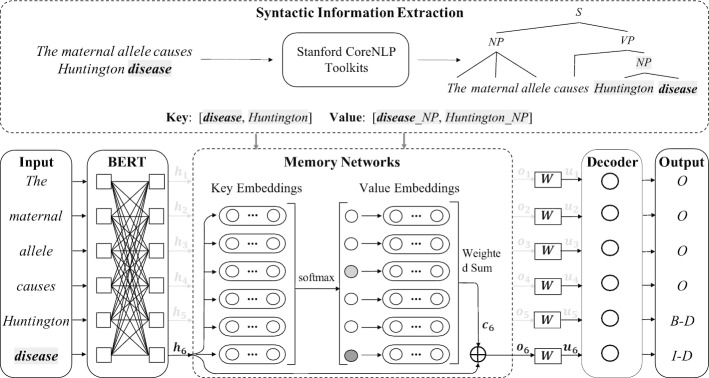
The overall architecture of BioKMNER. The top part of the figure shows the syntactic information extraction process: for the input word sequence, we firstly use off-the-shelf NLP toolkits to obtain its syntactic information (e.g., syntax tree), then map the context features and the syntactic information into keys and values, and finally convert them into embeddings. The bottom part is our sequence labeling based BioNER tagger, which uses BioBERT [19] as the encoder and a softmax layer as the decoder. Between the encoder and decoder are the key-value memory networks (KVMN) which weighs syntactic information (values) according to the importance of the context features (keys). The output of KVMN is fed into the decoder to predict output labels

The overall architecture of our BioKMNER is shown in Fig. 3. Following the common approaches in BioNER, we treat it as a sequence labeling task, where the input word sequence ${\mathcal {X}}=\{x_{1}, x_{2}, \ldots , x_{i}, \ldots x_{l}\}$ is tagged with a sequence of NE labels $\widehat{\mathcal {Y}}=\{{\widehat{y}}_{1}, {\widehat{y}}_{2}, \ldots , {\widehat{y}}_{i}, \ldots {\widehat{y}}_{l}\}$. In our method, we propose key-value memory networks (KVMN) [26] to incorporate syntactic information. Specifically, context features and their carrying syntactic information instances are mapped to keys and values in KVMN, where the values are weighed according to the comparison between the keys and the input words.

In this section, we firstly introduce the syntactic information extraction process. Then we elaborate the KVMN module used to incorporate the syntactic information. Finally, we explain how our NER method works with the KVMN module.

### Syntactic information extraction

**Fig. 4:**
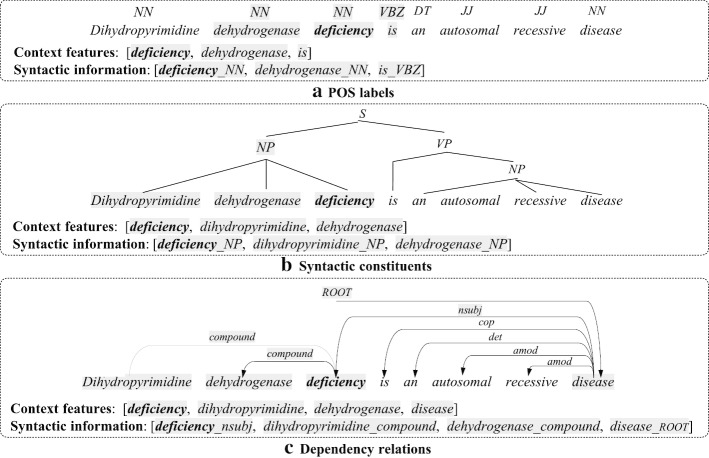
Syntactic information extraction. Three types of syntactic information extracted for an example “Dihydropyrimidine dehydrogenase deficiency is an autosomal recessive disease” in the biomedical domain. The context features and their corresponding POS labels, syntactic constituents, and dependency relations for $x_5$=“deficiency” are highlighted in part **a**, **b**, and **c** respectively

In our study, we focused on three types of syntactic information: POS labels, syntactic constituents, and dependency relations. To obtain such information, we first run the off-the-shelf NLP toolkits on the input sentence ${\mathcal {X}}$. Then for each word $x_{i}$ in ${\mathcal {X}}$, we extract the context features associated with $x_{i}$ and their corresponding syntactic information instances. Figure 4 shows the three types of context features and their corresponding syntactic information instances^12^ for the sentence “Dihydropyrimidine dehydrogenase deficiency is an autosomal recessive disease”.^13^ This figure focuses on the word “deficiency” (in boldface) with its highlighted context features and their corresponding syntactic information.

*POS labels* Given a current word $x_{i}$ in ${\mathcal {X}}$, we use a 1-word window to extract the context words and their POS labels at both sides of $x_i$. As is shown in Fig. 4a, for word “deficiency”, the context features are [deficiency, dehydrogenase, is] and the corresponding syntactic instances are [deficiency_*NN*, dehydrogenase_*NN*, is_*VBZ*].

*Syntactic constituents*

First, we define a set of acceptable syntactic nodes (denoted by $\mathcal {L}$) which contains 10 different constituent types^14^ to selected syntactic constituents from the syntax tree of the input ${\mathcal {X}}$. Then, for each word $x_{i}$ in ${\mathcal {X}}$, we start with the leaf of $x_{i}$ in the parse tree, search up to find the first acceptable syntactic node which is in $\mathcal {L}$. After finding the first acceptable node of $x_{i}$, the words under that node and their combination with the node type label are selected as the context features and their corresponding syntactic information respectively. As is shown in Fig. 4b, for word “deficiency”, the first acceptable node is *NP*, and there are three words under this NP span. So the context features are [deficiency, dihydropyrimidine, dehydrogenase], and the syntactic instances are [deficiency_*NP*, dihydropyrimidine_*NP*, dehydrogenase_*NP*].

*Dependency relations* According to the dependency relations of the words in the sentence, we first collect the dependents and the governor of the given word (i.e., first-order dependency relations). Then, we regard its dependents, its governor, and the word itself, as the context features and regard the combination of these words and their dependency types as the syntactic instances. In Fig. 4c, for the given word “deficiency”, it has two dependents (i.e., “dihydropyrimidine” and “dehydrogenase”) and one governor (i.e., “disease”, which is the root of the sentence). According to these dependency relations, the context features of “deficiency” are [deficiency, dihydropyrimidine, dehydrogenase, a, metabolic] and the syntactic information instances are [deficiency_*nsubj*, dihydropyrimidine_*compound*, dehydrogenase_*compound*, disease_*ROOT*].

Through these processes, the context feature list $\mathcal {K}$ and the syntactic instance list $\mathcal {V}$ are built upon the extraction results for each type of syntactic information. For each word ${x}_{i}$ in the word sequence ${\mathcal {X}}$, in both training and predicting process, associated context features and syntactic information instances in $\mathcal {K}$ and $\mathcal {V}$ are activated and computed. We denote the context features and the syntactic information instances for $x_i$ as $\mathcal {K}_{i}=[k_{i,1}, \ldots , k_{i,j}, \ldots k_{i,m_{i}}]$ and $\mathcal {V}_{i}=[v_{i,1}, \ldots , v_{i,j}, \ldots v_{i,m_{i}}]$, respectively. Note that the context feature list $\mathcal {K}$ and syntactic instance list $\mathcal {V}$ used in our model do not necessarily need to include all three types of the syntactic information discussed above. In other words, our model can leverage each type of syntactic information independently. In the following subsection, we illustrate our method to leverage the keys and values through KVMN.

### The memory module

Previous methods to leverage syntactic information for BioNER are limited in concatenating the embeddings of syntactic information instances with the input word embeddings. This method fails to distinguish the useful syntactic instances in a specific context, so that noisy syntactic information may hurt model performance. Therefore, we propose to use KVMN to enhance the incorporation process of syntactic information. Originally, KVMN is firstly proposed to incorporate the information in a list of memory slots $(k_{j}, v_{j})$ (where $k_{j}$ and $v_{j}$ refer to keys and values, respectively)^15^ into a model for question answering tasks. In KVMN, it addresses the keys by assigning a probability weight to the value in each memory slot by comparing the question (which is denoted as *x*) to each key:

1 $$\begin{aligned} p_j = softmax ( {\mathbf {A}} \Phi _X (x) \cdot {\mathbf {A}} \Phi _K (k_j)) \end{aligned}$$

where $\Phi _{\cdot }$ are feature mapping matrices and ${\mathbf {A}}$ is a matrix. Then, KVMN reads the values by computing the weighted sum using the resulting probability weights:

2 $$\begin{aligned} {\mathbf {o}} = \sum _{j} \ p_j \cdot {\mathbf {A}} \Phi _V (v_j) \end{aligned}$$

Afterwards, ${\mathbf {o}}$ is incorporated into the question representation by an element-wise summation: ${\mathbf {o}}'= {\mathbf {A}} \Phi _X (x) + {\mathbf {o}}$ and the resulting ${\mathbf {o}}'$ is used to predict the answers of the question. Therefore, in KVMN, the keys are used to compute the weights, which is used to address the values with respect to the input; the values are used to incorporate useful information into the input presentation and thus improve model performance. Considering that knowledge base entries have been used as a possible type of resources for the memory slots to incorporate extra knowledge into the input representation by transforms between the keys and values [26], we can also use such transforms between context features and syntactic information instances to incorporate the syntactic information into our backbone method. In doing so, not only is the syntactic information addressed by comparing the input with context features (which we think is more intuitive than comparing the input with syntactic information), but also different syntactic information instances are weighed according to the comparison between keys and the input, which allows our method to distinguish the important syntactic information instances and leverage them accordingly.

In our approach to bioNER, we adapt the KVMN to a sequence labeling paradigm by applying it to each word $x_{i}$ in the input. Therefore, for $x_{i}$, its hidden vector ${\mathbf {h}}_{i}$ obtained from an encoder serves as the counterpart of input representation ${\mathbf {A}} \Phi _X (x)$; its associated context features and the corresponding syntactic information instances stand for the keys $k_{j}$ and values $v_{j}$, respectively. In details, the memory module takes $\mathbf {{h}_{i}}$ for each ${x}_{i}$, activates the keys to address their embeddings and computes the probability weights for them by

3 $$\begin{aligned} p_{i, j} = \frac{exp\left( \mathbf {{h}_{i}} \cdot {\mathbf {e}}^{k}_{i,j}\right) }{\sum _{j=1}^{m_{i}} exp\left( \mathbf {{h}_{i}} \cdot \mathbf {e^{k}_{i,j}}\right) } \end{aligned}$$

where ${\mathbf {e}}^{k}_{i,j}$ is the embedding vector of $k_{i.j}$. Afterwards, we use the resulting probabilities on syntactic information instances in $\mathcal {V}_{i}$ to get the weighted value embedding ${\mathbf {o}}_{i}$:

4 $$\begin{aligned} {\mathbf {o}}_{i} = \sum _{j=1}^{m_{i}} p_{i,j} {\mathbf {e}}^{v}_{i,j} \end{aligned}$$

where ${\mathbf {e}}^{v}_{i,j}$ is the embedding vector of the value $v_{i,j}$. Once ${\mathbf {o}}_{i}$ is obtained for each ${x}_{i}$, we concatenate^16^ it with $\mathbf {{h}_{i}}$ to get the ${\mathbf {o}}'_{i}$, which can be represented by ${\mathbf {o}}'_{i}=\mathbf {{h}_{i}} \oplus \mathbf {{o}_{i}}$.

### Tagging with KVMN

To facilitate the process of leveraging syntactic information through KVMN, we firstly use an encoder to obtain the hidden vector ${\mathbf {h}}_i$ for each $x_{i}$. Among different types of encoders, in our method, we use the prevailing BioBERT [19], which is demonstrated to be an effective encoders for many biomedical NLP tasks, such as relation extraction [22] and natural language inference [46]. Therefore, the process to obtain the hidden vectors for the input ${\mathcal {X}}$ can be represented by

5 $$\begin{aligned}{}[{\mathbf {h}}_{1}, {\mathbf {h}}_{2}, \ldots , {\mathbf {h}}_{i}, \ldots , {\mathbf {h}}_{l}] = BioBERT({\mathcal {X}}) \end{aligned}$$

Once ${\mathbf {o}}'_{i}$ is obtained from the KVMN module, we apply a trainable matrix ${\mathbf {W}}$ to it to align its dimension to the output space, which is formalized by

6 $$\begin{aligned} \mathbf {{u}_{i}}={\mathbf {W}} \cdot {\mathbf {o}}'_{i} \end{aligned}$$

The resulting vector ${\mathbf {u}}_{i}$ is a weight vector with each dimension corresponding to a type of BioNER labels (so its vector dimension matches the number of NE types). Finally, we apply a softmax function to ${\mathbf {u}}_{i}$ to predict the output label ${\widehat{y}}_{i}$ for ${x}_{i}$ by

7 $$\begin{aligned} {\widehat{y}}_{i} = \arg \max \frac{exp(u^{t}_i)}{\sum _{t=1}^{|{\mathcal {T}}|} exp(u^{t}_{i})} \end{aligned}$$

where ${\mathcal {T}}$ refers to the label set and $u^{t}_{i}$ is the value at dimension *t* in the weight vector ${\mathbf {u}}_{i}$.

## Data Availability

The datasets generated and/or analysed during the current study are available in the *BioKMNER* repository https://github.com/cuhk-nlp/BioKMNER. The code is available at https://github.com/cuhk-nlp/BioKMNER.

## Abbreviations

BioNER: Biomedical named entity recognition
KVMN: Key-value memory networks
NER: Named entity recognition
NE: Named entity
POS: Part-of-speech

## Footnotes

1 https://biocreative.bioinformatics.udel.edu/tasks/biocreative-ii/task-1a-gene-mention-tagging/.

2 http://www.geniaproject.org/shared-tasks/bionlp-jnlpba-shared-task-2004.

3 https://biocreative.bioinformatics.udel.edu/tasks/biocreative-v/track-3-cdr/.

4 https://pubmed.ncbi.nlm.nih.gov/.

5 https://github.com/spyysalo/standoff2conll.

6 https://github.com/spyysalo/s800.

7 We use v3.9.2, downloaded from https://stanfordnlp.github.io/CoreNLP/.

8 We obtain the pre-trained models v1.1 from https://github.com/naver/biobert-pretrained

9 We evaluate all models by the widely used *seqeval* framework at https://github.com/chakki-works/seqeval.

10 All experiments are run on a single Nvidia Tesla V100 GPU with 16G RAM.

11 https://spacy.io/.

12 We combine the word and its syntactic information to increase the diversity of the syntactic information instance.

13 The POS tags and parse trees are obtained from running the online demo of Stanford CoreNLP Toolkits [25] at https://corenlp.run/.

14 The 10 accepted constituent types are $\textit{NP}$, $\textit{VP}$, $\textit{PP}$, $\textit{ADVP}$, $\textit{SBAR}$, $\textit{ADJP}$, $\textit{PRT}$, $\textit{INTJ}$, $\textit{CONJP}$ and $\textit{LST}$, which are selected from the types used in the CoNLL-2003 shared task [41].

15 Here, we use the subscript *j* instead of *i* in the original paper to avoid confusion, because *i* is already used to refer to the input word $x_i$ at the position *i*.

16 We use concatenation instead of element-wise summation in KVMN, which does not change the nature of using KVMN.
